# Epigenetic regulation of the circadian clock: role of 5-aza-2′-deoxycytidine

**DOI:** 10.1042/BSR20170053

**Published:** 2017-05-19

**Authors:** Tatsunosuke Tomita, Ryoji Kurita, Yoshiaki Onishi

**Affiliations:** Biomedical Research Institute, National Institute of Advanced Industrial Science and Technology (AIST), DBT-AIST International Laboratory for Advanced Biomedicine (DAILAB), Higashi 1-1-1, Tsukuba 305-8566, Japan

**Keywords:** circadian clock, DNA methylation, gene induction, hematological malignancy

## Abstract

We have been investigating transcriptional regulation of the *BMAL1* gene, a critical component of the mammalian clock system including DNA methylation. Here, a more detailed analysis of the regulation of DNA methylation of *BMAL1* proceeded in RPMI8402 lymphoma cells. We found that CpG islands in the *BMAL1* and the *PER2* promoters were hyper- and hypomethylated, respectively and that 5-aza-2′-deoxycytidine (aza-dC) not only enhanced *PER2* gene expression but also *PER2* oscillation within 24 h in RPMI8402 cells. That is, such hypermethylation of CpG islands in the *BMAL1* promoter restricted *PER2* expression which was recovered by aza-dC within 1 day in these cells. These results suggest that the circadian clock system can be recovered through *BMAL1* expression induced by aza-dC within a day. The *RPIB9* promoter of RPMI8402 cells, which is a methylation hotspot in lymphoblastic leukemia, was also hypermethylated and aza-dC gradually recovered *RPIB9* expression in 3 days. In addition, methylation-specific PCR revealed a different degree of aza-dC-induced methylation release between *BMAL1* and *RPIB9*. These results suggest that the aza-dC-induced recovery of gene expression from DNA methylation is dependent on a gene, for example the rapid response to demethylation by the circadian system, and thus, is of importance to clinical strategies for treating cancer.

## Introduction

Circadian rhythms function in most living organisms and govern many behavioral and biochemical processes with 24-h periodicity, regardless of changes in the cellular environment. The master clock that generates circadian rhythms in mammals is located in the suprachiasmatic nucleus (SCN) of the hypothalamus. The master clock is governed by blue-light sensing in the eye and it controls all the aspects of physiology such as sleep-wake cycles, body temperature, hormone secretion, blood pressure and metabolism [1]. The molecular mechanism of the circadian oscillator is based on interlocked transcriptional/translational feedback loops that have both positive and negative elements. The circadian oscillator orchestrates the output of the rhythmic mRNA expression of typically hundreds or thousands of clock-controlled genes (CCGs) that are mediated by transcription factors or coregulators with rhythmic abundance. Whereas post-transcriptional regulation contributes to the rhythmic transcription of mature abundant mRNA, transcriptional regulation remains the dominant determinant of the rhythmic transcriptome [2].

Transcriptional regulation initially requires the coordinated control of chromatin and the genome structure [3]. In general, genetic information is packed into the chromatin structure, and the nucleosome is the most basic unit of the chromatin structure; it determines the large-scale chromatin structure as a building block and influences transcription. Eukaryotic promoter regions are thought to have inactive states, assured by the tendency of nucleosomes to inhibit transcription by protecting protein–DNA interaction. Therefore, chromatin remodeling and loosening the nucleosomal barrier including histone tail modifications are key steps in circadian modifications. For example, rhythmic BMAL1/CLOCK binding, histone H3 Lys4 trimethylation (H3K4me3) and Lys9 acetylation are required as well as rhythmic H3 abundance at the start site for *Dbp* transcription [4]. In addition, protein complexes containing clock proteins such as PER contain various interactive partners with known catalytic activity towards chromatin [5,6].

The methylation of cytosines on CpG dinucleotides [7], which is also epigenetic regulation of gene expression, either directly interferes with the binding of transcriptional regulators, or indirectly inactivates a gene by modulating chromatin to a repressive structure. Light-induced DNA methylation is dynamic at specific promoters that correspond to circadian gene expression in the SCN [8] and altered DNA methylation is associated with many human diseases. *Clock* gene methylation is highly prevalent in dementia with Lewy bodies (DLB), a disorder that is similar to Parkinson’s disease [9], in which the *NPAS2* promoter is hypomethylated [10]. DNA methylation is also prevalent in various types of cancer and clock genes influence tumorigenesis; for example the methylation of *clock* gene promoters such as *CLOCK* [11] and *PERs* [12–15] contribute to cancer progression. Many tumor suppressors and oncogenes are under circadian control and *Per* genes function as tumor suppressors [16].

Amongst the core clock genes, *BMAL1* expression oscillates in the SCN and in peripheral clock cells, this is closely associated with circadian rhythms [17]. The hypermethylation of CpG islands in the promoter of *BMAL1* transcriptionally silences its expression in hematological malignancies [18,19]. We previously found that REV-ERB orphan nuclear receptors (ROREs), which are recognition motifs for ROR and REV-ERB orphan nuclear receptors and critical elements for *BMAL1* oscillatory transcription [20], are embedded in a unique GC-rich open chromatin structure, with which a nuclear matrix like structure at the 3′-flanking region co-operates to regulate *BMAL1* transcription [21,22]. We also found that DNA demethylation of the *BMAL1* promoter in CPT-K cells enhances *BMAL1*, and then *PER2* and *CRY1* transcription, and finally circadian functions is recovered [19]. The present study further investigates the effects of DNA demethylation in detail.

## Materials and methods

### Chemicals

The premix reagent for real-time quantitative PCR was SYBR(R) Premix Ex Taq (TM) II (Tli RNaseH Plus) from Takara Bio (Shiga, Japan). Reverse transcription proceeded using the PrimeScript™ RT Reagent Kit with gDNA Eraser from Takara Bio, according to the manufacturer’s instructions. D-luciferin potassium salt was purchased from Wako (Osaka, Japan). All other chemicals were of reagent grade and used without further purification.

### Cell culture

RPMI8402 cells [23] were cultured in Dulbecco’s modified Eagle’s medium (DMEM) supplemented with 10% FBS and a mixture of penicillin and streptomycin in a humidified incubator at 37°C under a 5% CO_2_ atmosphere.

### CpG methylation analysis

We identified CpG islands in *PER2, BMAL1*, and *RPIB9* promoters using the algorithm at www.urogene.org/methprimer [24]. Methylation was analyzed as a modification generated using EpiTect Bisulfite from Qiagen (Hilden, Germany) according to the manufacturer’s instructions, followed by PCR cloning and sequencing. The primer sequences were as follows: *BMAL1*: 5′-GTGTGGTTTGGGTATTGTAGTGG-3′ and 5′-CACATCAAACAAAATTCTTC-3′; *PER2*: 5′-GGTGTTGTTATTTTTTTTTGGGTTG-3′ and 5′-CCAACAACCCCAAAAAACTTCC-3′; *RPIB9*: 5′-GTGAGTGTTAGAGGATTTGATTTAAGTTGG-3′ and 5′-AACCACCCACACTCCACAACCACTC-3′.

### Promoter assay

A luciferase reporter gene plasmid containing the *PER2* promoter [25] and the internal control plasmid, pRL-CMV from Promega (Madison, WI, U.S.A.) were transfected into RPMI8402 cells using Lipofectamine and Plus reagents (Thermo) according to the manufacturer’s instructions. Reporter luciferase activities were measured using the Dual Luciferase Reporter Assay System (Promega) as described [26]. Transcriptional activities were normalized relative to *Renilla* luciferase activities.

### Real-time quantitative RT-PCR

Reverse transcription of total RNA in each cell lysate was performed using PrimeScript™ RT Reagent Kit described above according to the manufacturer’s instructions. These obtained reactants were applied to real-time quantitative PCR. The real-time quantitative PCR is performed with LightCycler® Nano(R) from Roche (Basel, Switzerland) and SYBR(R) Premix Ex Taq (TM) II (Tli RNaseH Plus; Takara Bio) as described [23]. The primer sequences were as follows: *PER2*: 5′-TGATTGAAACCCCAGTGCTCGT-3′ and 5′-CTCCATGGGTTGATGAAGCTGG-3′; *BMAL1*: 5′-AGGACTTCCCCTCTACCTGCTC-3′ and 5′-AACTACATGAGAATGCAGTCGTC-3′; *RPIB9*: 5′-GGCCAGTCACAAGAAGGAGA-3′ and 5′-GAGATCCACAGAGGCCAAGT-3′; *ACTIN*: 5′-TACGCCAACACAGTGCTGTCTG-3′ and 5′-TTTTCTGCGCAAGTTAGGTTTTGTC-3′. All individual PCR products were confirmed using polyacrylamide electrophoresis and cloned into the pGEM-T Easy vector (Promega) to prepare an authentic template. Relative expression levels were evaluated using LightCycler® software, version 3.5 (Roche).

### Real-time reporter gene assays

Real-time reporter gene assays were proceeded as described [21]. RPMI8402 cells transfected with the *PER2* reporter plasmid were stimulated with 50% FBS for 2 h and then incubated with DMEM containing 0.1 mM luciferin (Promega), 25 mM HEPES (pH 7.2) and 10% FBS. Bioluminescence was measured and integrated for 1 min at 10-min intervals using Kronos (R)Dio A2550 from ATTO Corporation (Tokyo, Japan). Data were detrended by subtracting a best fit line followed by subsequent fitting to a sine wave to determine the length of the circadian period as described [27].

### Quantitative analysis of DNA methylation

Cytosine methylation in total genomic DNA was quantitated using MethylFlash Methylated DNA Quantification Kits from EpiGentek (Farmingdale, NY, U.S.A.) according to the manufacturer’s instructions. Briefly, 100 ng of genomic DNAs from RPMI8402 cells were immobilized in microtiter plates, washed twice and incubated with 50 μl of anti-methylcytosine antibody (1 µg/ml) at 37°C for 1 h. The mixture was then incubated with 50 μl of horseradish peroxidase-labeled secondary antibody (400 ng/ml) at 37°C for 1 h. Color was developed by incubation with 50 μl of 3,3′,5,5′-tetramethylbenzidine for 30 min at room temperature and the reaction was stopped with 50 μl of 2 N HCl. Absorbance at 450 nm was analyzed using a microplate reader (Bio–Rad, model 680). Methylated dCTP weight and ratio in genomic DNA was determined from a linear slope between methylcytosine and absorbance in a range from 0 to 2 ng.

### Methylation-specific PCR

Genomic DNA modified with bisulfite as described above served as a template for methylation-specific PCR. The primer sequences designed using MethPrimer [24] were as follows: methylated *BMAL1*: 5′-GGGATTTAGAGAAGAGGGATATTTC-3′ and 5′-AATCATTTAACGCACAAAAACG-3′; unmethylated *BMAL1*: 5′-GGGATTTAGAGAAGAGGGATATTTT-3′ and 5′-CAATCATTTAACACACAAAAACACA-3′; methylated *RPIB9*: 5′-TTTGAGGGAGTAGTTTAGTTGGATC-3′ and 5′-TACCAATATAAAATCTTTTCGCGTC-3′; unmethylated *RPIB9*: 5′-TTTGAGGGAGTAGTTTAGTTGGATT-3′ and 5′-TACCAATATAAAATCTTTTCACATC-3′.

The PCR products cloned into the pGEM-T Easy vector (Promega) served as an authentic template. The PCR products were quantitated by real-time PCR proceeded using a LightCycler® Nano(R) (Roche) and SYBR(R) Premix Ex Taq™ II (Tli RNaseH Plus (Takara Bio) as described [21]. Expression was evaluated using LightCycler® software, version 3.5.

$$\text{Unmethylated ratio }=\frac{\text{Unmethylated amount}}{\text{Unmethylated amount }+\text{methylated amount}}$$

## Results

### Aza-dC releases methylation of BMAL1 CpG islands

We previously reported that ROREs in the *BMAL1* promoter are embedded in a unique GC-rich open chromatin structure under CpG island hypomethylation, which is important for circadian transcription [21,22]. On the other hand, CpG islands in the *BMAL1* promoter of some cancer cell lines are hypermethylated and the *BMAL1* gene is transcriptionally silenced [19].

**Figure 1:**
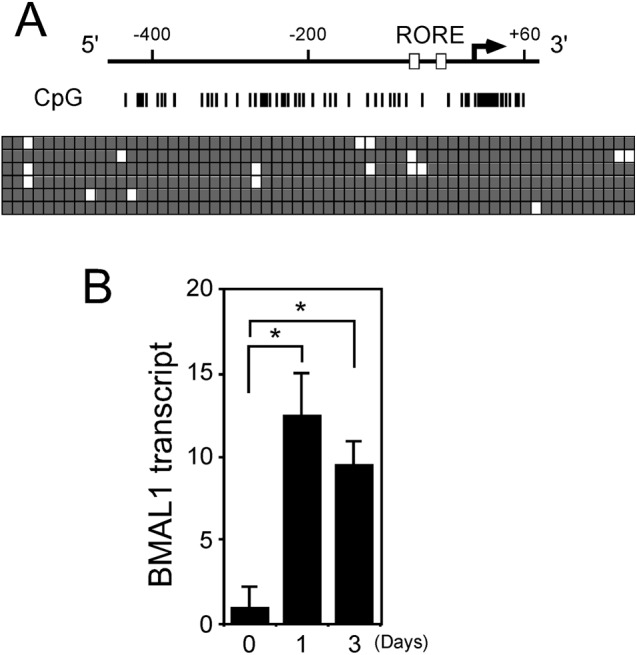
DNA hypermethylation of *BMAL1* gene in RPMI8402 cells (**A**) Hypermethylation of DNA in *BMAL1* promoter. *BMAL1* promoter sequence was modified with bisulphite and then CpG islands were analyzed. Vertical lines, CpG sites in *BMAL1* promoter region. Filled and unfilled squares, methylated and unmethylated CpG sites, respectively. Arrow and open boxes in map, transcription start site and two recognition motifs for ROR and REV-ERB orphan nuclear receptors (RORE), respectively. (**B**) Aza-dC activates *BMAL1* within 1 day. RPMI8402 cells were incubated with 2.5 µM aza-dC for indicated days, then RNA was analyzed by qRT-PCR. Levels of RNA were normalized to those of *ACTIN* expression and value for cells incubated without aza-dC was set at 1. Values are means ± SE of triplicate assays.*P<0.05; Student’s *t* test.

A survey of cells with hypermethylated CpG islands in the *BMAL1* promoter found that the human lymphoblastic leukemia cell line, RPMI8402, has methylated CpG islands. The bisulphite genomic sequencing of six individual clones indicated that the *BMAL1* promoter in RPMI8402 cells is hypermethylated in CpG islands (Figure 1A). We used RT-PCR to study the effects of 5-aza-2′-deoxycytidine (aza-dC) on suppressed *BMAL1* transcription in RPMI8402 cells to clarify its relationship with promoter methylation. Demethylation of the CpG islands in the promoter using aza-dC induced the transcription of *BMAL1* 13- and 9-fold at 1 and 3 days, respectively, in RPMI8402 cells (Figure 1B). These results suggested that hypermethylation of the promoter CpG islands represses *BMAL1* transcription in RPMI8402 cells and that the demethylation CpG islands in the promoter by aza-dC enhanced *BMAL1* transcription within 1 day.

### *PER2* gene expression profile in RPMI8402 cells

**Figure 2:**
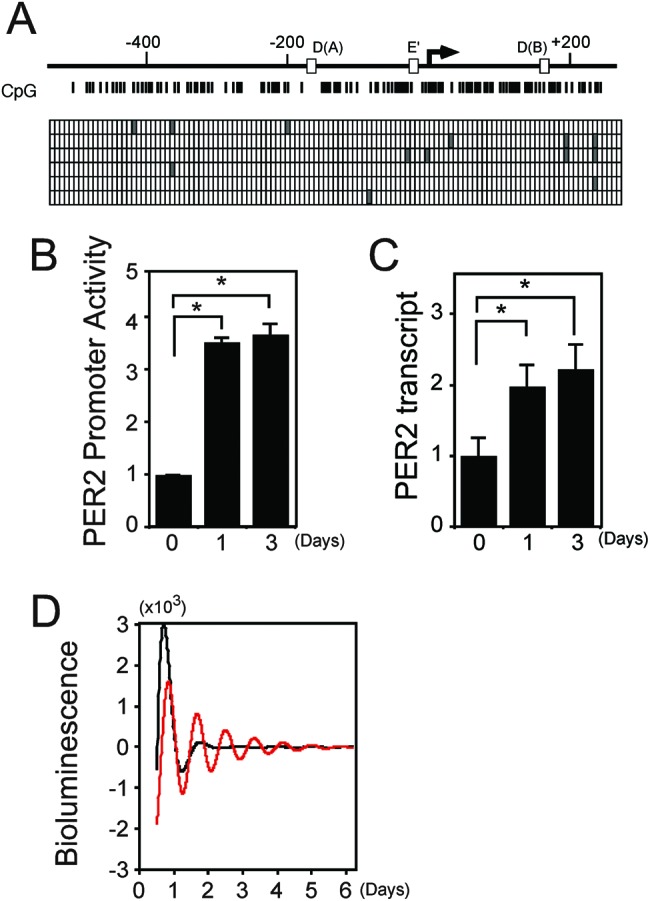
Aza-dC activates *PER2* gene expression at the level of transcription (**A**) Hypomethylation of DNA in *PER2* promoter. Genomic sequence of RPMI8402 cells was analyzed after modification with bisulphite. Vertical lines, CpG sites in *PER2* promoter region. Filled and unfilled squares, methylated and unmethylated CpG sites, respectively. Arrow, transcription start site; unfilled boxes D (A) and D (B), DBP-binding sites; unfilled box E’, non-canonical E-box. (**B**) Aza-dC activates *PER2* promoter within 1 day. Transcription assays proceeded using construct containing *PER2* promoter with 2.5 µM aza-dC. Normalized expression levels were calculated relative to luciferase activities in cells incubated without aza-dC. Values are means ± SE of triplicate assays. *P<0.05; Student’s *t* test. (**C**) Aza-dC enhances *PER2* transcripts within 1 day. RPMI8402 cells were incubated with 2.5 µM aza-dC for indicated days, and then RNA was analyzed using qRT-PCR. Levels of RNA were normalized to those of *ACTIN* expression, and value for cells incubated without aza-dC was set at 1. Values are means ± SE of triplicate assays. *P<0.05; Student’s *t* test. (**D**) Aza-dC recovers transcriptional oscillation of *PER2*. RPMI8402 cells transfected with *PER2* reporter plasmid were stimulated with 50% FBS for 2 h, and then bioluminescence was measured in presence of 2.5 µM aza-dC. Detrended fit curves are representative of at least three independent experiments (control, gray; aza-dC, red).

We investigated the gene expression of *PER2*, a circadian clock component. We applied bisulphite sequencing to investigate the methylation profile of the *PER2* promoter as described above for *BMAL1*. The sequence results of six individual clones showed that a maximum of four cytosine bases were methylated in the CpG islands of the *PER2* promoter region (Figure 2A), indicating that the region is hypomethylated and quite different from that of the *BMAL1* promoter region. We also studied the effects of aza-dC on *PER2* promoter activity and transcription (Figure 2B,C, respectively). *PER2* promoter activity was 3.5- and 3.8-fold enhanced on days 1 and 3 compared with day 0 (Figure 2B), suggesting that the expressed *BMAL1* affected activation. The amount of *PER2* transcripts was 2.0- and 2.3-fold enhanced on days 1 and 3 compared with day 0 (Figure 2C), indicating over a two-fold increase over the basal value. These results indicated that although the CpG region of the *PER2* promoter was hypomethylated, aza-dC enhanced *PER2* transcription in RPMI8402 cells.

### Aza-dC recovers circadian oscillation of the *PER2* gene

The expression of *BMAL1* was restricted in RPMI8402 cells as described above and the *BMAL1* function released by aza-dC reflected activities of other genes, such as *PER2* transcription. We therefore investigated the oscillation of *PER2* transcription using a *PER2*-Luc real-time reporter assay system. In the absence of aza-dC, the reporter proceeded with the first induction, but the oscillation then became damped (Figure 2D, gray line). In the presence of aza-dC, the reporter oscillated robustly for over 5 days (period length: 23.3 h; Figure 2D, red line). These results imply that aza-dC can regenerate endogenous circadian rhythms through restoration of *BMAL1* expression and *PER2* induction in RPMI8402 cells.

### Aza-dC recovers *RPIB9* expression

**Figure 3:**
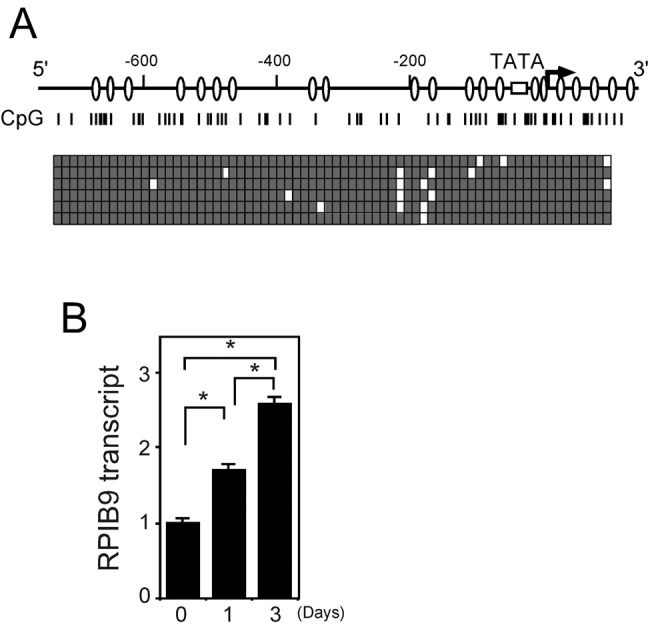
Effects of aza-dC on *RPIB9* promoter, a methylation hotspot in leukemic cells (**A**) Hypermethylation in *RPIB9* promoter. *RPIB9* promoter sequence was modified with bisulphite and then CpG islands were analyzed. Vertical lines, CpG sites in *RPIB9* promoter region. Filled and unfilled squares, methylated and unmethylated CpG sites, respectively. Arrow, open box and ovals in map, transcription start site, TATA box and putative SP-1 binding regions, respectively. (**B**) Aza-dC gradually activates *RPIB9*. RPMI8402 cells were incubated with 2.5 µM aza-dC for indicated days, and then RNA was analyzed using qRT-PCR. Levels of RNA were normalized to those of *ACTIN* expression, and value for cells incubated without aza-dC was set at 1. Values are means ± SE of triplicate assays.*P<0.05; Student’s *t* test.

The *RPIB9* (*RUNDC3B*) gene is a candidate of a biomarker in lymphoid malignancy which possibly serves as a mediator between Rap2 and the MAPK signaling cascade [28]. Studies have indicated that the *RPIB9* gene is methylated in acute myelogenous leukemia (AML) and malignant B cells and that aza-dC enhances the expression of this gene [29,30]. We examined the methylation status of *RPIB9* in RPMI8402. The DNA sequences of six individual clones (Figure 3A) showed that CpG islands in the *RPIB9* promoter region are hypermethylated. We applied real-time quantitative RT-PCR to determine the effects of aza-dC on *RPIB9* transcription. Figure 3B shows that *RPIB9* expression gradually increased 1.8- and 2.5-fold on days 1 and 3, respectively, after aza-dC demethylation compared with the amount of mRNA on day 0. These results indicate that aza-dC gradually changed the transcriptional profile of *RPIB9* and that this profile is quite different from those of clock genes.

### Aza-dC releases methylation in *BMAL1* and *RPIB9* promoters at different rates

**Figure 4:**
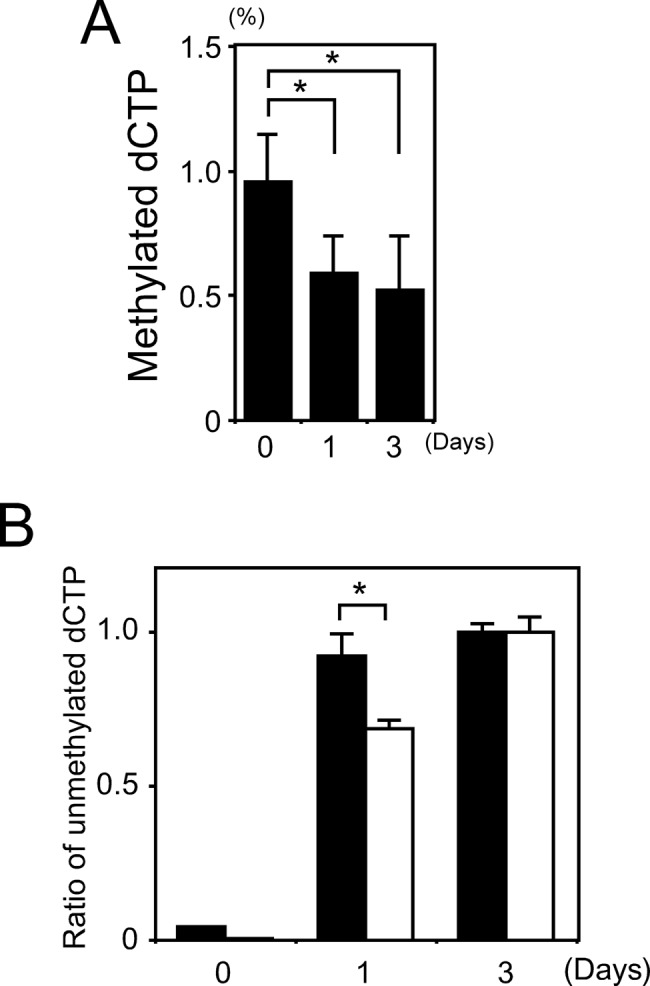
Effects of aza-dC on methylation of genome in RPMI8402 cells (**A**) Methylcytosine of genomic DNA in RPMI8402 cell. Amounts of methylcytosine were determined using anti-methylcytosine antibody as described in ‘Materials and methods’ section and indicated as methylated dCTP ratios by weight in genomic DNA (%). Values are means ± SE of triplicate assays. *P<0.05; Student’s *t* test. (**B**) Demethylation in *BMAL1* completes earlier than that in *RPIB9*. Filled and unfilled boxes show demethylation ratios of *BMAL1* and *RPIB9*, respectively. Genomic DNA prepared from RPMI8402 was incubated with 2.5 µM aza-dC for indicated days, modified with bisulfite and analyzed using methylation-specific PCR. Value for cells incubated without aza-dC for 3 days was set at 1. Values are means ± SE of triplicate assays.*P<0.05; Student’s *t* test.

We compared the total genomic content of methylated dCTP with and without aza-dC to determine the effects of aza-dC on methylcytosine in the genomic DNA of RPMI8402 cells. Figure 4A shows that approximately 1% of the total dCTP was methylated in the absence of aza-dC, which was consistent with previous findings [31,32] whereas the amount of methylated dCTP was reduced by approximately half within 1 day in the presence of aza-dC.

We then quantitated the unmethylated ratios of CpG islands in the promoter regions of both *BMAL1* and *RPIB9* in the presence and absence of aza-dC. Figure 4B shows that the islands in both *BMAL1* and *RPIB9* promoters were methylated without aza-dC, because their unmethylated ratios were quite low at 0.04 and 0.003, respectively. However, aza-dC demethylated almost all CpG islands in the *BMAL1* promoter (Figure 4B, black bars) and approximately 70% of those in the *RPIB9* promoter within 1 day (Figure 4B, white bars). These results indicate that the rate of demethylation of the CpG islands was slower in *RPIB9* than in *BMAL1*, suggesting that the demethylation mechanisms differ amongst these genes.

## Discussion

Changes in the methylation of clock gene DNA cause the misregulation of various critical cell physiological processes that can lead to diseases such as various types of cancers [33]. For example, the circadian oscillation of gene expression is aberrant in leukemic cells, as *BMAL1, PER1*, and *PER2* are down-regulated in patients with chronic lymphocytic leukemia [34]. Disrupted *CRY2* and *PER2* are associated with non-Hodgkin’s lymphoma [18] and the initiation and/or progression of AML [35], respectively, and CpG islands of *PER3* are highly methylated in all patients with chronic myelogenous leukemia [36]. Taniguchi et al. [18] and we reported that *BMAL1* is epigenetically inactivated in hematologically malignant cells [19]. The findings of these reports together indicate that hematopoietic cell malignancies are associated with down-regulation of the circadian clock. used in this cell lines. We found hypermethylated CpG islands in the *BMAL1* promoter and repressed *BMAL1* expression (Figure 1) and hypomethylated CpG islands in the *PER2* promoter of human lymphoblastic RPMI8402 cells (Figure 2A). The expression of *BMAL1* (Figure 1B) and *PER2* (Figure 2B,C) as well as oscillation or function (Figure 2D) were recovered by aza-dC which is an anticancer drug [37]. These findings imply an association between the anticancer function of aza-dC and induction of the tumor-suppressor function of *PER2* [16]. These also indicate that methylation of the *BMAL1* promoter is a key factor in the oscillation of the clock gene, *PER2*. The down-regulation of clock genes might cause the up-regulation of typical oncogenes such as *c-Myc* and *Cyclin-D1* [34] that are both under the control of circadian genes. Therefore, these genes are likely to be aberrant in malignant cells with defective clock genes. On the other hand, a recent study has shown that disruption of the core circadian clock in a mouse model of AML causes antileukemic effects in AML (that is, *BMAL1* and *CLOCK* are necessary for AML cell growth) [38], suggesting that the mechanisms of aberrant clock gene expression in leukemia are highly complex. Further investigation at different stages of malignancy or in various types of leukemia are required to determine the function of clock genes in leukemia.

The most common epigenetic modification is DNA methylation, which is a covalent chemical modification that plays a crucial role in numerous biological processes. Generally, although approximately 70% of CpG sequences in the entire mouse and human genomes are methylated, CpG islands in promoter sequences are methylated at a relatively lower level. However, CpG islands of the promoter regions are frequently hypermethylated and expression of the corresponding gene is damped in tumor cells, a situation that is rather tumor type specific [39]. This implies that methylation status would be a good biomarker of malignant stage in specific tissues and cells. The non-CpG methylation of DNA can regulate gene expression through affecting the binding of transcription factors [40]. Bisulphite-based methods are the most prevalent means of distinguishing between cytosine and 5′-methylcytosine in epigenetic studies. For example, bisulphite-sequencing, combined bisulphite restriction analysis (COBRA), methylation specific PCR and pyrosequencing can identify the methylation status of a specific sequence at the level of a single CpG. On the other hand, quantitative analysis of global DNA methylation is difficult because a limitation is that bisulphite-based methods are inherently prone to variability due by DNA degradation caused by the required acidic conditions [41]. We therefore quantitated global cytosine methylation using an immunochemical approach that does not require either bisulfite or methylation-sensitive enzymes, suggesting that DNA degradation is minimized and that this method of quantitation is highly accurate. Assays using anti-methylcytosine antibody (Figure 4A) indicated a 1% global methylation rate in the genomic DNA of RPMI8420 cells, which was consistent with previous findings [31,32] and that aza-dC reduced this by approximately 0.5%, indicating the release of DNA methylation. Such information could serve as a biomarker of cancer prognosis.

The level of DNA methylation within a ±1 kb region surrounding the transcription start site closely correlated with gene repression [42]. The hypermethylation of CpG islands surrounding the transcription start site (Figures 1 and 3) indicated that the *BMAL1* and *RPIB9* genes are repressed, which is in agreement with the above. The methylation of DNA promotes stable nucleosome positioning of methylated CpG dinucleotides in the minor groove in proximity to the histone octamer complex [43] and those in the major groove influence nucleosome dynamics towards a more open structure [44]. We previously described an open chromatin structure in the promoter region of *BMAL1* with hypomethylated CpG [21,22], suggesting a lesser effect of DNA methylation on nucleosome positioning at the *BMAL1* promoter region. DNA methylation affects the binding dynamics of transcription factors and knocking out DNA methyltransferases increases the number of binding events of the transcription factor NRF1 [45]. Besides, the methylation of CpG adjacent to the core Sp1 motif decreases the Sp1/Sp3 binding [46], which might be related to the repression of *BMAL1* transcription by DNA methylation because many putative Sp1 binding motifs are located around the *BMAL1* promoter [47]. The mechanism of the repression by DNA methylation remains unclear and further studies are required.

Figure 4B shows that recovery from DNA methylation by aza-dC differs between the *BMAL1* and *RPIB9* genes, suggesting that the release of methylation depends on the locus/gene or sequence. Taken together with the quantitative results of global methylation (Figure 4A), the demethylation rate of *BMAL1* was essentially comparable with that of other genes, whereas that in *RPIB9* was slower (Figure 4B), suggesting that methylation status is DNA site specific. One of the most important issues regarding DNA methylation is how the machinery is directed towards and maintains specific sequences in the genome. One answer might be the PML-RAT fusion protein in leukemia, which induces DNA hypermethylation and gene silencing at specific target promoters [48]. siRNA-mediated, RNA-directed DNA methylation is a stepwise process initiated by dsRNAs that recruit DNMT to catalyze the *de novo* DNA methylation of specific regions [49]. Therefore, although the susceptibility of individual CpG islands to *de novo* methylation might intrinsically differ, the mechanism remains obscure. The methylation of CpG is strictly regulated and stable, and changes in methylation profiles are associated with diseases including cancer, indicating close relationships amongst biological function, DNA methylation sites and the mechanism of methylation. Taken together, these findings imply that methylation is specific to gene function and an early response to the aza-dC demethylation of sites in *BAML1* might be functionally important for adaptation to environmental changes. The information herein provides novel insights into clock gene function that should affect the clinical treatment and diagnosis of diseases.

## Abbreviations

aza-dC: 5-aza-2′-deoxycytidine
DMEM: Dulbecco’s modified Eagle’s medium
RORE: ROR and REV-ERB orphan nuclear receptor responsive element
SCN: suprachiasmatic nucleus
